# Attenuation of Inflammatory Symptoms by Icariside B2 in Carrageenan and LPS-Induced Inflammation Models via Regulation of MAPK/NF-κB Signaling Cascades

**DOI:** 10.3390/biom10071037

**Published:** 2020-07-11

**Authors:** Md Badrul Alam, Yoon-Gyung Kwon, Shakina Yesmin Simu, Sk Abrar Shahriyar, Sang Han Lee

**Affiliations:** 1 School of Food Science and Biotechnology, Graduate School, Kyungpook National University, Daegu 41566, Korea; mbalam@knu.ac.kr (M.B.A.); yongyeung@knu.ac.kr (Y.-G.K.); 2 Food and Bio-Industry Research Institute, Inner Beauty/Antiaging Center, Kyungpook National University, Daegu 41566, Korea; 3 Department of Brain Science, Ajou University School of Medicine, Suwon 16499, Korea; simu90@aumc.ac.kr; 4 Biomedical Science, Graduate School, Ajou University School of Medicine, Suwon 16499, Korea; sksy.kmu@gmail.com; 5 knu BnC, Kyungpook National University, Daegu 41566, Korea

**Keywords:** anti-inflammatory effects, icariside B2, NF-κB signaling, MAP-kinase

## Abstract

Prolonged inflammatory responses can lead to the development of several chronic diseases, such as autoimmune disorders and the development of natural therapeutic agents is required. A murine model was used to assess the anti-inflammatory effects of the megastigmane glucoside, icariside B2 (ICSB), and the assessment was carried out in vitro, and in vivo. The in vitro anti-inflammatory effects of ICSB were tested using LPS-stimulated BV2 cells, and the protein expression levels of inflammatory genes and cytokines were assessed. Mice were subcutaneously injected with 1% carrageenan (CA) to induce acute phase inflammation in the paw. Inflammation was assessed by measuring paw volumes hourly; subsequently, the mice were euthanized and the right hind paw skin was expunged and processed for reverse transcription-polymerase chain reaction (RT-PCR) and Western blot analyses. ICSB inhibits LPS-stimulated nitric oxide (NO) and prostaglandin E2 (PGE_2_) generation by reducing the expression of inducible NO synthase (iNOS) and cyclooxygenase 2 (COX-2). ICSB also inhibits the COX-2 enzyme with an IC50 value of 7.80 ± 0.26 µM. Molecular docking analysis revealed that ICSB had a strong binding affinity with both murine and human COX-2 proteins with binding energies of −8 kcal/mol and −7.4 kcal/mol, respectively. ICSB also reduces the manifestation of pro-inflammatory cytokines, such as TNF-α, IL-6, and IL-1β, at their transcriptional and translational level. ICSB hinders inhibitory protein κBα (IκBα) phosphorylation, thereby terminating the nuclear factor kappa-light-chain-enhancer of activated B cell (NF-κB) nuclear translocation. ICSB also represses the mitogen-activated protein kinases (MAPKs) signaling pathways. ICSB (50 mg/kg) showed an anti-edema effect in CA-induced mice and suppressed the CA-induced increases in iNOS and COX-2 protein levels. ICSB attenuated inflammatory responses by downregulating NF-κB expression through interference with extracellular signal-regulated kinase (ERK) and p38 phosphorylation, and by modulating the expression levels of iNOS, COX-2, TNF-α, IL-1β, and IL-6.

## 1. Introduction

Microglia are specialized macrophages of the central nervous system (CNS) that play a vital role in both CNS homeostasis and immune defense. In the brain, the inflammatory reaction is deeply coupled with microglial activation, demonstrated by numerous indications, such as the synthesis and secretion of pro-inflammatory cytokines and chemokines and the breakdown of the blood–brain barrier [1]. Chronic and excessive microglial activation can develop severe neurodegenerative diseases, such as Alzheimer’s disease, Parkinson’s disease, multiple sclerosis, and amyotrophic lateral sclerosis [2]. Consequently, there is a growing demand to discover and develop new therapeutic agents targeting active microglia and neuroinflammation.

Carrageenan (CA)-induced inflammation is possible in a highly reproducible and well-established mouse model [3,4], characterized by the immediate progress of edema, with the secretion of various early phase inflammatory mediators, such as histamine, serotonin, and bradykinin. Prostaglandins emerge during the late phase of inflammation. Increasing levels of TNF-α, IL-1β, and IL-6 also contribute to this inflammation [4,5]. The development of paw edema was maximal at approximately 4–5 h post-CA injection and is viewed as an immediate inflammatory response, which is decreased by the action of inhibitors within the inflammatory cascade. Therefore, this model has and will continue to have a vital role in drug development.

The mitogen-activated protein kinases (MAPKs), such as extracellular signal-regulated kinase (ERK), the p38 mitogen-activated protein kinase (p38 MAPK), and the c-Jun NH2-terminal kinase (JNK), are a group of signaling proteins—their phosphorylation is acknowledged as a critical component for the generation of pro-inflammatory cytokines in activated microglia, and they also play a vital role in neuroinflammation [6,7]. Increasingly, research has focused on pinpointing natural molecules that are safe and can prevent inflammatory disease by impeding upstream signaling events associated with inflammatory gene expression. 

Epimedium (Berberidaceae) is believed to “nurture the kidney and boost Yang” and has a long history of use in traditional Chinese medicine. Now, *Epimedium brevicornu* Maxim., *E. sagittatum* (Sieb. and Zucc.) Maxim., *E. pubescens* Maxim., *E. koreanum* Nakai., and *E. wanshanense* T. S. Ying are recorded as the official sources of Herba Epimedii in the Chinese Pharmacopoeia [8]. A renowned botanical supplement, which uses extracts from the aerial parts of these plants, and has been widely used in China, Japan and Korea for more than 2000 years against sexual dysfunction, osteoporosis, cardiovascular diseases, menstrual irregularity, asthma, chronic nephritis, and immunoregulation [9,10]. In the last few decades, the chemical and pharmacological actions of the plants of Epimedium have drawn extensive interest; more than 270 secondary metabolites have been identified from the species of Epimedium [11]. Cumulative evidence has revealed that Epimedium can be used to treat osteoporosis, climacteric period syndrome, breast lumps, hyperpiesia, and coronary heart disease. Epimedium also has immunity-enhancing, anti-aging, anti-tumor, and anti-AIDS pharmacological activities [8,11]. Consequently, Epimedium sp. has enormous potential for research and exploitation as it possesses various anti-inflammatory activities, whereas the anti-inflammatory mechanistic studies of icariside B2 (ICSB) have been poorly understood. In this study, we investigate the anti-inflammatory effects and molecular mechanisms of ICSB in LPS-induced microglial cells and CA-induced inflammatory mice that influence MAPK signaling pathways. 

## 2. Materials and Methods

### 2.1. COX-2 Enzyme Inhibiton

The COX-2 enzyme inhibition capability of ICSB was checked out using a Cayman colorimetric COX (ovine) inhibitor screening assay kit (Catalog number 560101, Cayman Chemical, Ann Arbor, MI, USA) according to the manufacturer’s instructions. Briefly, the different concentrations of ICSB (1, 2, 5, 10, 15, 30, 50, and 100 µM) and indomethacin, used as positive control, were mixed with a reaction mixture, containing 1.0 mM hematin, 2.0 mM phenol, and 5 mM EDTA and an ovine purified COX-2 in 0.1 M Tris-HCl buffer (pH 8.0), and preincubated for 5 min at room temperature. Then, the reaction was started by adding arachidonic acid (5.0 mM) for 20 min at 37 °C. The reaction was stopped by the addition of 1 M HCl. The absorbance was measured using an ELISA reader ‘‘rainbow’’ (TECAN, Switzerland). The percentage (%) inhibition was calculated using the following equation: {[PGE_2_]vehicle − [PGE_2_]drug)/([PGE_2_]vehicle} × 100.

### 2.2. Cell Viability and Nitric Oxide Determination

The murine BV2 microglial cells (ATCC, Rockville, MD, USA) were maintained in Dulbecco’s Modified Essential Medium (DMEM), supplemented with 100 units/mL penicillin–streptomycin, and 10% heat-inactivated fetal bovine serum (FBS) (Grand Island, NY, USA) in a humidified atmosphere of 5% CO_2_ at 37 °C. Cell viability was assessed by MTT assay to define the cell toxicity of ICSB. Briefly, BV2 cells at a density of 1 × 10^5^ per well were cultured in a 96-well plate for 24 h, treated with different concentrations of ICSB (25, 50, and 100 μM) or vehicle alone for 2 h and were then co-cultured with LPS (500 ng/mL) at 37 °C for an additional 20 h. Following incubation, the cell-free culture mediums (100 µL) were collected for NO production by the Griess assay, and cell viability was measured as previously described [12].

### 2.3. TNF-α, IL-1β, IL-6, and PGE_2_ Assays

BV2 cells (2 × 10^5^ cells/mL) were cultured for 20 h, treated with a predetermined concentration of ICSB (25, 50, and 100 μM) for 1 h, followed by co-culturing with 500 ng/mL LPS at 37 °C for an additional 12 h. Subsequently, an enzyme-linked immunosorbent assay (ELISA) was performed for the quantification of TNF-α, IL-1β, IL-6, and PGE_2_, adhering to the manufacturer’s protocol (Invitrogen, Frederick, MD, USA).

### 2.4. CA-Induced Paw Edema

The Institute of Cancer Research (ICR) mice (strain: IcrTac:ICR, albino, eight-weeks-old) were taken from Central Lab Animals, Inc. (Seoul, Korea) and housed in a well-defined, air-conditioned animal room (temp: 23 ± 1 °C, humidity: 55 ± 5%, and 12 h light/dark cycle) with ad libitum access to water and a standard laboratory diet. Following an acclimatization period of 1 week, animals were randomly divided into five groups (*n* = 6): the negative control group (G1); the CA-induced control group (G2); the indomethacin group (G3); the 25 mg/kg/day ICSB group (G4); the 50 mg/kg/day ICSB group (G5). ICSB at doses of 25 and 50 mg/kg/day were administered to the mice (p.o.) for four consecutive days. At day five, 1% CA in saline (60 μL per animal) was injected subcutaneously into the right hind paw of each mouse to induce acute inflammation 30 min after ICSB or vehicle administration. Indomethacin, an anti-inflammatory drug, was used as a positive control. Paw volumes were measured hourly for 4 h after injection using a plethysmometer (UGO BASILE; Comerio, VA, USA). The mice were then euthanized and the right hind paw skin was collected and immediately frozen in a nitrogen tank for Western blotting analysis. The experiment was conducted in accordance with the guidelines for animal experiments issued by Kyungpook National University and approved by the Institutional Animal Care and Use Committee of Kyungpook National University (KNU-2018-0025).

### 2.5. Transfection and Luciferase Assays for NF-kB

BV2 cells were transfected with a pNF-κB-Luc reporter gene (Beyotime Biotechnology, Nantong, China) for 24 h, followed by ICSB treatment for 2 h. Subsequently, they were co-cultured with or without LPS for another 8 h. Cell lysates were prepared for measuring luciferase activity using the Luciferase Assay System (Promega, Madison, WI, USA).

### 2.6. mRNA Analysis by a Semi-Quantitative Reverse Transcriptase-Polymerase Chain Reaction

BV2 cells were pretreated with ICSB (25, 50, and 100 μM) for 30 min and then co-cultured with LPS (500 ng/mL) for 20 h to estimate mRNA levels. Total RNA was extracted by TRIzol^®^ Reagent (Invitrogen Co., Carlsbad, CA, USA), complying with the manufacturer’s protocol. Semi-quantitative RT-PCR reactions were conducted by using the respective primer described in Supplementary Table S1, as previously reported [13]. PCR products were visualized by ethidium bromide staining after electrophoresis; the bands were analyzed using the Image Lab™ Software, version 5.2.1 (Bio-Rad Laboratories, CA, USA).

### 2.7. Immunofluorescence Assay

The BV2 microglial cells were cultured directly on sterile glass coverslips in 24-well plates for 24 h, pretreated with ICSB for 1 h with or without LPS, fixed in 4% paraformaldehyde for 20 min at room temperature, permeabilized with 0.1% Triton X-100 in PBS, and blocked with 3% BSA. Afterwards, the cells were sequentially incubated with rabbit NF-κB(p65) antibody (Santa Cruz Biotechnology Inc., Eugene, OR, USA) at room temperature and Alexa 546-labeled goat anti-rabbit IgG (Molecular Probes, Eugene, OR, USA) at room temperature for 1 h. A Hoechst staining solution (Hoechst 33258) was used to visualize the cell nucleus. After washing with PBS, the samples were mounted with Vectashield mounting medium (Vector Laboratories, Burlingame, CA, USA), using glass coverslips, and visualized under a fluorescence microscopy (Leica Micosystems, DM2500, Wetzlar, Germany).

### 2.8. Western Blotting Analysis

BV2 cells (2 × 10^5^ cells/mL) were treated with a predetermined concentration of ICSB (25, 50, and 100 μM) for 1 h, followed by being co-cultured with 500 ng/mL LPS at 37 °C for an additional 1 h for phospho-IκBα (phospho-inhibitory protein IκBα) and IκBα, p-p38, p-JNK, and p-extracellular signal-regulated kinase (p-ERK) and 20 h for cyclooxygenase-2 (COX-2), inducible nitric oxide synthase (iNOS0), and nuclear factor kappa-light-chain-activated B cells (NF-κB). All anti-bodies information used in this study was described in Supplementary Table S2. A nuclear and cytoplasmic extraction kit (Sigma-Aldrich Co., St. Louis, MO, USA) was used to prepare nuclear and cytosolic protein extracts. SDS-PAGE separated an aliquot of 30 µg total protein, and then proteins were electro-transferred to nitrocellulose membranes (Whatman GmbH, Dassel, Germany), blocked with 5% skim milk in TBST buffer, and blotted with each primary antibody (1:1000) and its corresponding secondary antibody (1:5000) [14]. An ECL solution system (Perkin Elmer) was utilized to detect the antigen–antibody reaction. The Image Lab™ Software, version 5.2.1 (Bio-Rad Laboratories, CA, USA) was used to visualize and analyze the band intensity [14].

### 2.9. Molecular Docking Study

The X-ray crystallographic structure of human and mouse COX-2 proteins (Protein Data Bank: 5IKQ and 1CVU, respectively) were obtained from the Protein Data Bank (PDB) (http://www.rcsb.org). All non-receptor atoms, such as water, ions, and miscellaneous compounds, were removed to prepare the proteins for docking using a UCSF Chimera 1.13.1 (http://www.cgl.ucsf.edu/chimera). The co-crystallized inhibitors/substrates were employed to define the corresponding active site, the flexible residues within the active site, and a validation criterion of docking calculations (re-docking). An MMFF94 force was applied to prepare the ligand (ICSB) with minimal energy using ChemBio3D Ultra, version 12.0 (PerkinElmer, Waltham, MA, USA). The molecular docking simulations were performed using AutoDock Vina (The Scripps Research Institute, La Jolla, CA, USA) [15]. The resolution of the protein structure, the center of the grid box, and the dimensions are described in Supplementary Table S3. The lowest binding energy was considered as the best docking conformation and was visualized using a PyMOL Molecular Graphics System (version 1.7.4, Schrödinger, Inc., New York, NY, USA). The 3D and 2D binding conformations were visualized as diagrams using Discovery Studio Visualization, version 4.5 (Accelrys, Inc., San Diego, CA 92121, CA, USA) and LigPlot viewer (EMBL-EBI, Wellcome Genome Campus, Hinxton, Cambridgeshire, CB10 1SD, UK, http://www.ebi.ac.uk/thornton-srv/software/LIGPLOT).

### 2.10. Statistical Analysis

All data were analyzed by one-way analysis of variance (ANOVA), followed by Dunnett’s multiple-comparisons test using SigmaPlot (SigmaPlot, Ver 12.5, Systat Software, Inc., Chicago, IL, USA), and are expressed as the mean ± SD (*n* = 4). A value of *p <* 0.05 was considered significant.

## 3. Results

### 3.1. ICSB as a Major Ingredient in E. koreanum Nakai

**Figure 1:**
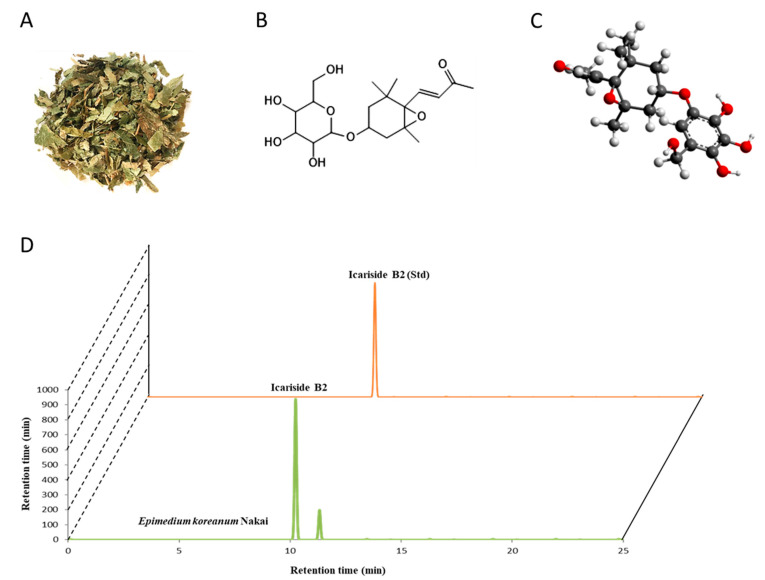
The classical photograph of the leaves of *E. koreanum* Nakai (**A**). 2D (**B**) and 3D (**C**) chemical structure of Icariside B2 (ICSB), a megastigmane glucoside identified by HPLC analysis (**D**) in the leaves of *E. koreanum* Nakai.

Epimedium has enormous potential for research and exploitation. Contained within Epimedium sp. is ICSB, a megastigmane derivative. In this report, we confirmed *E. koreanum* Nakai, as shown in Figure 1A possessed ICSB, as shown in Figure 1B,C, as a principal ingredient in its extract, which was confirmed by HPLC analysis, as shown in Figure 1D.

### 3.2. Effect of ICSB on the Production of Inflammatory Mediators in LPS-Induced BV2 Cells

**Figure 2:**
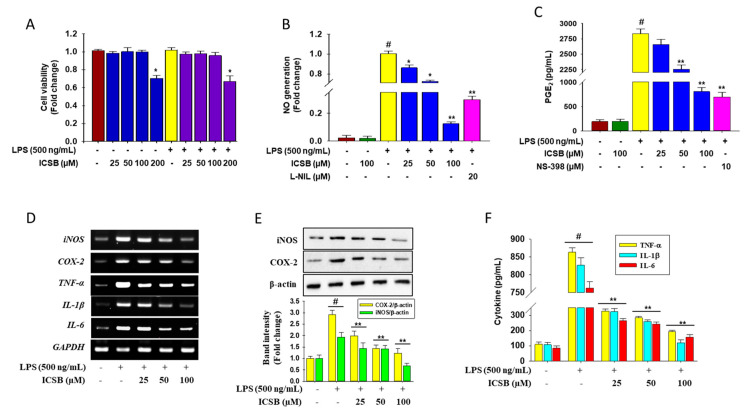
Inhibition of the production of inflammatory and pro-inflammatory mediators by ICSB in LPS-induced BV2 cells. Cells (2 × 10^5^ cells/mL) were treated with various concentrations (25, 50, or 100 μM) of ICSB for 1 h and then incubated with LPS (500 ng/mL). Cell toxicity effect of ICSB (**A**) and inhibitory effects of ICSB on LPS-induced production of NO (**B**) and PGE_2_ (**C**) in BV2 cells. mRNA expression iNOS, COX-2, TNF-α, IL-1β, and IL-6 (**D**); protein expression of iNOS and COX-2 (E); production of pro-inflammatory cytokines (F) in LPS-induced BV2 cells. Results are the mean ± S.D. of three separate experiments. # *p* < 0.01 is significant compared with vehicle-treated control; * *p* < 0.05 and ** *p* < 0.01 are significant compared with LPS alone.

Figure 2A shows that the experimental doses of ICSB (25, 50, and 100 μM) did not have any toxic effect on BV2 cells, whereas ICSB at 200 μM showed cellular toxicity. As shown in Figure 2B, LPS treatment significantly increased the cellular NO generation (column 3) compared with untreated cells (column 1), whereas ICSB treatment significantly reduced NO generation in a dose-dependent manner (columns 4–6). ICSB (100 µM) and L-NIL, specific iNOS inhibitors (20 µM), suppressed the LPS-stimulated NO generation by 8.3-fold and 3.4-fold, respectively (columns 6 and 7). Figure 2C shows the effect of ICSB on the generation of PGE_2_, shown in Figure 2B. As shown in Figure 2C, LPS treatment significantly increased PGE_2_ production (column 3) compared with untreated cells (column 1), whereas ICSB treatment significantly suppressed PGE_2_ production in a concentration-dependent fashion (columns 4–6). In contrast, NS-398, a selective COX-2, halted the production of LPS-induced PGE_2_ 3.7-fold.

RT-PCR analysis revealed that the mRNA expression of iNOS and COX-2 was amplified markedly by LPS treatment, whereas ICSB treatment halted this trend in BV2 cells, as shown in Figure 2D. As expected, the pretreatment of ICSB also significantly alleviated the protein level of iNOS and COX-2 in a dose-dependent manner in BV2 cells, as shown in Figure 2E. These data indicate that ICSB had the potential to suppress the LPS-induced production of NO and PGE_2_ by regulating their respective genes, iNOS and COX-2, in activated microglial cells.

Figure 2D shows that the mRNA expression of pro-inflammatory cytokines TNF-α, IL-1β, and IL-6, was increased noticeably by LPS treatment 5.0-, 4.6- and 4.2-fold, respectively, whereas ICSB treatment ceased this trend in BV2 cells in a dose dependent manner. Interestingly, ICSB, at 100 µM, successively suppressed the mRNA expression of TNF-α, IL-1β, and IL-6 2.1-, 2.6- and 1.7-fold, respectively, in LPS-stimulated BV2 cells. As expected, ELISA analysis demonstrated that ICSB pretreated cells significantly lessened the cellular production of TNF-α, IL-1β, and IL-6 in a dose-dependent manner, as shown in columns 3–5 of Figure 2F, than that of LPS-treatment only, as shown in column 2 of Figure 2F. These results suggest that ICSB could suppress the LPS-induced production of TNF-α, IL-1β, and IL-6 through the downregulation of the respective genes in activated microglial cells.

### 3.3. Effect of ICSB on Upstream Signaling for NF-κB Activation in LPS-Induced BV2 Cells

**Figure 3:**
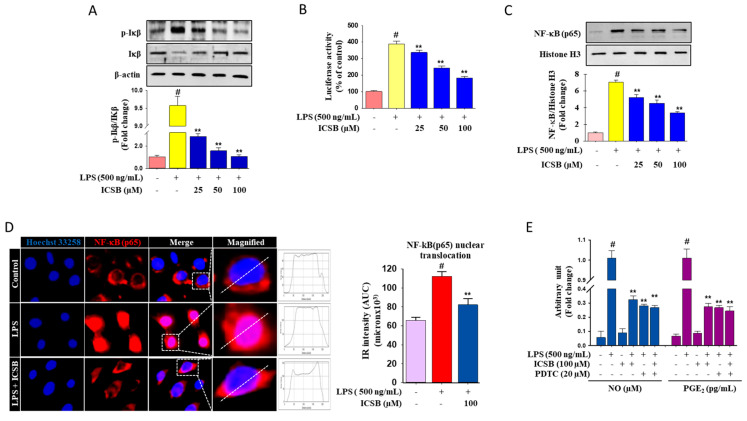
Effects of ICSB on NF-κB signaling. BV2 cells were treated with ICSB in the presence or absence of LPS (500 ng/mL) for 20 h. Effects of ICSB on IκB phosphorylation (**A**), in LPS-stimulated BV2 cells by Western blotting. The quantification of relative band intensities from three independent experimental results was determined by densitometry. ICSB inhibits LPS-induced promoter activity of NF-κB in BV2 cells (**B**). The pNF-κB-luc, containing four NF-κB binding motifs (GGGAATTTCC) and pRL-SV40 reporter constructs, were transiently transfected into BV2 cells. Following different treatment, the promoter activity was detected using the Dual-Luciferase^®^ Reporter Assay System. ICSB inhibits the LPS-induced nuclear translocation of NF-κB in BV2 cells (**C**). Immunofluorescent staining was performed to detect the cellular distribution of NF-κB(p65) (red). Hoechst 33258 (blue) was used to stain the nuclei Fluorescence intensity profile for NF-κB(p65) across a transverse section of one cell is presented adjacent to the magnified image. The dotted line shows the cross section of the single cell for the fluorescence intensity profile. Quantification for the relative IR intensity (AUC) for the NF-κB(p65) nuclear translocation is presented in the adjacent graph. Nuclear translocation of NF-κB(p65) IR intensity was measured for eight randomly selected cells from each group with ImageJ (**D**). ICSB inhibits the production of NO and PGE_2_ with or without PDTC, a pharmacological inhibitor of NF-κB in LPS-stimulated BV2 cells (**E**). Results are the mean ± S.D. of three separate experiments. # *p* < 0.01 is significant compared with vehicle-treated control; * *p* < 0.05 and ** *p* < 0.01 are significant compared with LPS alone.

Figure 3A indicates that LPS insult noticeably increases the IκB phosphorylation level 9.48-fold, which was reduced by ICSB treatment in a dose-dependent fashion. In contrast, the total IκBα protein levels were not restored. LPS treatment boosted the transcriptional activity of NF-κB 3.9-fold in activated microglia, whereas the dose-dependent treatment of ICSB pointedly down-regulated the activation of NF-κB, as shown in Figure 3B. Furthermore, immunoblot analysis demonstrated that the nuclear translocation of NF-κB (p65) dramatically increased 6.5-fold after LPS exposure, when compared with untreated cells, as shown in column 2 of Figure 3C, which was considerably blocked by ICSB treatment 2.4-fold, as shown in columns 3–5 of Figure 3C. As expected, as shown in Figure 3D, immunofluorescence microscopic analysis revealed that pretreatment of ICSB was significantly suppressed the LPS-stimulated nuclear translocation of NF-κB(p65). by. To validate the anti-inflammatory effects of ICSB via the regulation of the NF-κB signaling cascade, PDTC, a pharmacological inhibitor of NF-κB was applied to determine the cellular generation of NO and PGE_2_ after LPS insult in BV2 cells. PDTC efficaciously suppressed LPS-induced cellular NO and PGE_2_ generation in activated microglial cells, as shown in Figure 3E. These results signified the prospective role of NF-κB in the probable mechanics of ICSB in regulating NO, PGE_2_, and pro-inflammatory cytokines in activated microglia cells.

### 3.4. ICSB Attenuated MAPK Phosphorylation in LPS-Stimulated BV2 Cells

**Figure 4:**
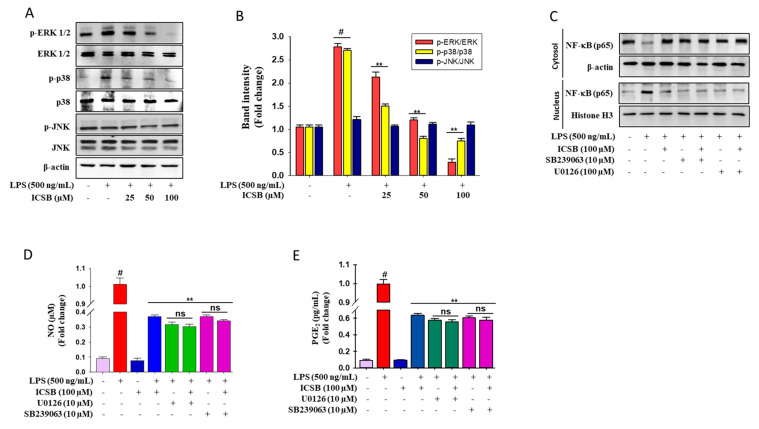
Effects of ICSB on MAPKs and NF-κB. (**A**) Cells (2 × 10^5^ cells/mL) were seeded in a 6-well plate and incubated for 24 h. Then, the cells were treated with indicated concentrations of ICSB (25–100 µM) or vehicle alone, followed by LPS (500 ng/mL), and were incubated for 1 h. (**A**) Effects of ICSB on the phosphorylation of mitogen activated protein kinases (MAPKs), c-Jun N-terminal kinases (JNK), extracellular signal-regulated kinases (ERKs), and p38 mitogen-activated protein kinases (p38) in LPS-stimulated BV2 cells. (**B**) Densitometric analysis of relative band intensities of proteins. Effect of ICSB on the nuclear translocation of NF-κB, (**C**) and the production of cellular NO generation (**D**) and PGE_2_ production (**E**) in the presence of ERK and p38 inhibitors. # *p* < 0.01 as compared with vehicle-treated control; ** *p* < 0.05 as compared with LPS alone.

As shown in Figure 4A,B, LPS exposure specifically triggered the phosphorylation of ERK1/2, and p38, whereas it had no effect on JNK phosphorylation. Pretreatment with ICSB considerably prevented the LPS-induced phosphorylation of p38 and ERK in a dose-dependent manner. Selective inhibitors U0126 and SB239063, of ERK and p38, respectively, were exploited to determine the NF-κB protein level in LPS-induced BV2 cells to explore whether the suppression of MAPK signaling lessens inflammatory symptoms. Interestingly, inhibitor treatment was successfully prevented the LPS-exposed nuclear translocation of NF-κB in activated BV2 cells, as shown in Figure 4C. These results suggest that the anti-inflammatory effect of ICSB might be correlated with the down-regulation of NF-κB signaling via the modulation of MAPKs phosphorylation in activated BV2 cells. In addition, to justify the effects of ICSB against inflammatory symptoms through the modulation of MAPKs function, ERK and p38 inhibitors were used to determine the cellular generation of NO and PGE_2_ after LPS insult in BV2 cells. Figure 4D,E show that both ERK (U0126) and p38 (SB239063) inhibitors effectively blocked LPS-induced cellular NO generation and PGE_2_ production in activated microglial cells, respectively.

### 3.5. Inhibitory Effects of ICSB on CA-Induced Mouse Hind Paw Edema

**Figure 5:**
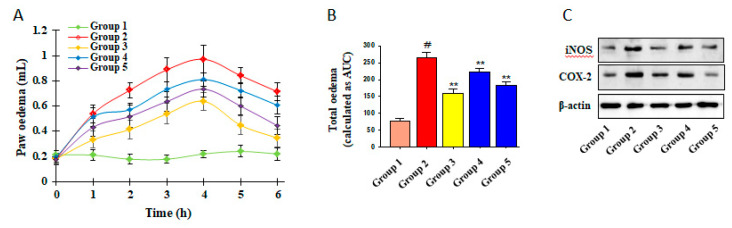
Inhibition of CA-induced paw edema by ICSB. (**A**,**B**) Paw volumes were measured 0–4 h after carrageenan injection, as described in materials and methods. Results represent mean ± S.D. of six animals. # *p* < 0.01 is significant compared with vehicle-treated control; * *p* < 0.05 and ** *p* < 0.01 are significant compared with CA alone by one way ANOVA, followed by a post-hoc Dunnett test. (**C**) Protein expressions of iNOS and COX-2 in a CA-induced mouse model.

To evaluate the anti-inflammatory effects of ICSB in vivo, we used the CA-induced swelling of mouse paw edema, in which indomethacin was used as a positive control. Indomethacin is a nonsteroidal anti-inflammatory drug (NSAID) and it works by inhibiting the production of prostaglandins through the inhibition of COX-2 and is commonly used as a prescription medication to reduce fever, pain, stiffness, and swelling from inflammation. As shown in Figure 5, CA-insult caused a significant increase in the swelling of the right hind paws of mice (G2) when compared with the control (G1). The pretreatment of indomethacin (G3; 10 mg/kg/day, p.o.), a positive control, as well as ICSB (G4 and G5: 25 and 50 mg/kg/day, p.o., respectively), considerably lowered paw edema formation, as shown in Figure 5A,B. Similarly, the pretreatment of ICSB drastically alleviated the iNOS and COX-2 protein expression, compared with the CA insult groups, as shown in Figure 5C. These data suggest that ICSB attenuated CA-induced inflammation in mice, plausibly through the suppression of iNOS and COX-2 protein.

### 3.6. Effect of ICSB on COX-2 Enzyme and Molecular Docking to the COX-2 Enzyme

**Figure 6:**
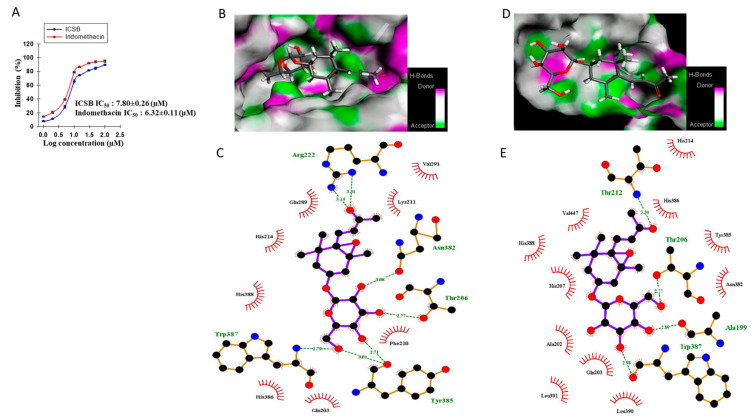
COX-2 enzyme inhibition and molecular docking analysis of ICSB with X-ray crystallographic structure of murine and human COX-2 protein (PDB: 5IKQ and 1CVU, respectively). COX-2 enzyme inhibition by ICSB (**A**). Bonding interaction between ICSB with murine COX-2 protein (PDB: 5IKQ) and human COX-2 protein (PDB: 1CVU) (**B**–**E**, respectively). The 3D and 2D binding conformation were visualized as diagrams using Discovery Studio Visualization, version 4.5 and LigPlot viewer.

In order to investigate whether ICSB could act as an anti-inflammatory agent, COX-2 enzyme inhibition as well as docking studies were implemented with both the human and mouse COX-2 enzyme (PDB: 5IKQ and 1CVU, respectively) with inbound inhibitor meclofenamic acid and arachidonic acid, respectively. As shown in Figure 6A, ICSB dose-dependently inhibits the COX-2 enzyme with an IC_50_ value of 7.80 ± 0.26 µM, whereas indomethacin has an IC_50_ value of 6.32 ± 0.11 µM. The molecular docking of the co-crystallized ligands for each enzyme with a low conformational RMSD value (<2.0 Å) indicated the validation of the present docking protocol, as shown in Supplementary Figure S1. The highest binding energies of ICSB were observed as −8 kcal/mol for murine COX-2 (PDB: 5IKQ) and −7.4 kcal/mol for human COX-2 (PDB: 1CVU); whereas those of co-bind inhibitor meclofenamic acid and arachidonic acid were −9 kcal/mol for murine COX-2 (PDB: 5IKQ) and −7.9 kcal/mol for human COX-2 (PDB: 1CVU), as shown in Supplementary Table S4. As shown in Figure 6B–E, the binding interaction between ligands and the COX-2 protein was varied, depending upon the ligand nature. ICSB interacted with the human COX-2 protein amino acid residues Arg222, Asn382, Gln203, Gln289, His214, His386, His388, Lys211, Phe210, Thr206, Trp387, Tyr385, and Val291, as shown in Figure 6B,C, while with murine COX-2 protein amino acid interacted with residues Ala 199, Ala 202, Asn382, Gln203, His207, His214, His386, His388, Leu390, Leu391, Thr206, Thr212, Trp387, Tyr385, and Val447, as shown in Figure 6D,E. However, the interaction of inbound ligand arachidonic acid showed binding outlines with Ala527, Gly526, Leu352, Leu359, Leu531, Leu534, Met113, Met522, Phe 381, Phe518, Ser353, Ser530, Trp387, Tyr 355, Tyr385, Val166, Val349, and Val523 amino acid residues, as shown in Supplementary Figure S2, of the human COX-2 protein, while meclofenamic acid of murine COX-2 was observed with Ala527, Gly526, Leu352, Leu531, Met522, Ser353, Ser530, Trp387, Tyr 348, Tyr385, and Val349 amino acid residue, as shown in Supplementary Figure S3.

## 4. Discussion

*Epimedium Koreanum* Nakai (Berberidaceae) has been used as a traditional Chinese medicine for the remedy of chronic inflammatory diseases, such as rheumatic arthritis and osteoporosis [8]. Recent scientific studies showed that the administration of Epimedium flavonoids improved learning and memory ability through the suppression of microglia and astrocyte activation, resulting in a decrease in the production of IL-1β and TNF-α in the hippocampus of an Alzheimer’s disease mouse model [16]. Moreover, cumulative studies revealed that megastigmane and its glucoside and glycoside derivatives had a potential to inhibit NO production in LPS-stimulated BV2 cells [17,18,19]. ICSB is a naturally occurring megastigmane glucoside found in various plants, including *Allium victorialis* var. *platyphyllum*, *Epimedium grandiflorum* MORR. var. *thunbergianum* (MIQ.) NAKAI., *Ficus hirta* Vahl., *Laurus nobilis* L., and *Erythronium japonicum*. The molecular action of ICSB against neuro-inflammatory symptoms has not been formally determined; therefore, evaluations of the anti-inflammatory properties of ICSB in LPS-exposed BV2 cells and CA-induced acute inflammatory mouse models were undertaken to investigate the mechanisms involved.

Inflammation is a multi-step biological response to toxic stimuli during the host’s defense process [20]. In particular, increased NO generation by iNOS has been described as cytotoxic molecules in inflammation and endotoxemia [21]. Numerous studies have indicated that megastigmane derivatives enact their anti-inflammatory effects through alleviating the production of NO, PGE_2_, TNF-α, and IL-1β via the down-regulation of the respective gene in murine macrophages [22,23,24]. In the present study, ICSB considerably inhibited the production of NO and PGE_2_, as well as downregulated their respective genes, iNOS and COX-2, after LPS insult in BV2 cells, as shown in Figure 2. Moreover, ICSB also inhibits the COX-2 enzyme dose-dependently, as shown in Figure 6A. These results demonstrate that ICSB might trigger iNOS and COX-2 inhibition, resulting in a decrease in NO and PGE_2_ production in LPS-stimulated microglial cells. Molecular docking analysis with COX-2 protein active site amino acid residues revealed that ICSB interacts with COX-2 proteins in other locations than the active site. It is well documented in the literature that COX-2 active sites possess three important regions. The first is a hydrophobic pocket, characterized by the presence of Tyr385, Trp387, Phe518, Ala201, Tyr248, and Leu352. The second is associated with three hydrophilic amino acid residues (Arg120, Glu524, and Tyr355), located at the entrance of the active site. The third is a side pocket, characterized by the presence of His90, Arg513, and Val523 [25]. The observed anti-inflammatory activity with meclofenamic acid and arachidonic acid in association with the interactive binding of COX-2 protein further denotes that the selected ICSB could be effectively used as an anti-inflammatory agent.

TNF-α and IL-1β are pro-inflammatory cytokines, crucially participating in the pathogenesis of inflammation [26]. IL-6 is also involved in several immunological responses and interacts with different target cells [27]. Our results revealed that ICSB treatment considerably reduced the mRNA expression and secretion of LPS-stimulated pro-inflammatory cytokines, as shown in Figure 2D,F, respectively. These results suggest that the inhibition of pro-inflammatory cytokines by ICSB might offer a novel means of curing neuro-inflammatory diseases.

NF-κB is one of the major transcriptional factors that can regulate the expression of iNOS, COX-2, and pro-inflammatory cytokines in inflammatory cells [28]. In a resting cell, IκB-α retains NF-κB in the cytoplasm by concealing the nuclear localization sequences on NF-κB subunits, and the phosphorylation of IκB causes the proteasomal degradation of IκB, increasing the nuclear localization of NF-κB, which activates various inflammatory and pro-inflammatory genes [6]. Many chemo-preventive and anti-inflammatory agents reduce inflammatory symptoms by suppressing NF-κB expression. Our results revealed that LPS exposure in microglial cells increased I-κBα phosphorylation and subsequently increased the nuclear translocation of NF-κB. However, ICSB treatment markedly reduced the phosphorylation of I-κBα, thereby hindering the nuclear translocation of NF-κB, as shown in Figure 3. These findings support the fact that ICSB treatment hinders NF-κB activation by overcoming the p-I-κBα level and nuclear translocation of NF-κB in LPS-induced BV2 cells. Our results demonstrate that ICSB successively inhibited the activation of NF-κB, which might be associated with the downregulation of iNOS, COX-2, TNF-α, IL-1β, and IL-6.

Several extracellular stimuli activate the MAPK family, which is composed of serine and threonine kinases and can regulate several signaling cascades within the cells. The intracellular signals, evolving from MAPK cascades, consistently activate a variety of secondary signaling molecules that ultimately lead to the activation of NF-κB [29]; thus, the inhibition of any or all of the three MAPKs is adequate to block the inflammatory response. Many megastigmanes have been reported to have anti-inflammatory properties by successively blocking the LPS-induced MAPKs phosphorylation [30,31]. However, the anti-inflammatory molecular mechanics of megastigmane depends on the substitution pattern in their core structure and the various target proteins [32]. The present study documents that ICSB significantly lowered the p-IκB-α level, inhibiting NF-κB nuclear translocation from the cytosol. Additionally, ICSB also prevented the phosphorylation of p38 and ERK in response to LPS stimulation. It was evident that ICSB mediated the inhibition of MAPKs, leading to the transcriptional inactivation of NF-κB, which, in turn, further downregulated COX-2 and iNOS expression and suppressed cytokine production. Our findings suggest that ICSB can modulate NF-κB directly via IκB modification or indirectly via MAPK inhibition.

CA is an important phlogistic agent, causing the induction of inflammatory responses, characterized by paw edema, neutrophil infiltration, capillary permeability, and the production of neutrophil-derived free radicals and mediators [33,34]. The CA-induced hind paw acute edematous inflammation animal model is generally used to distinguish the anti-inflammatory effects of candidate drugs [34]. Immediately after CA-insult, NO was produced through the stimulation of NOS, which is involved in the development of inflammatory symptoms [35]. According to our results, treatment with ICSB (25 and 50 mg/kg/day) resulted in a significant decrease in mouse paw edema volumes, as shown in Figure 5A,B. The treatment of ICSB also mitigated the expression of iNOS, and COX-2, in CA-induced mice, compared to that of the control, as shown in Figure 5C. These results suggest that the inhibitory mechanism of LPS-induced NO and PGE_2_ production by ICSB in BV2 cells may represent important molecular actions involved in the inhibition of the CA-induced formation of paw edema.

## 5. Conclusions

Collectively, we can conclude that ICSB is one of the active ingredients of *E. koreanum* Nakai, which mediates anti-inflammatory effects by downregulating NF-κB expression through interference with ERK and p38 phosphorylation. This study convincingly supports the potential of ICSB as a novel anti-inflammatory agent, whereas further systemic analyses are needed in other inducer-challenged animals to determine the in vivo efficacy and pharmacokinetics of ICSB.

## Supplementary Materials

The following are available online at https://www.mdpi.com/2218-273X/10/7/1037/s1, Table S1: List of the primer sets used in the study; Table S2: List of the primary antibodies used in the study; Table S3: The X-ray crystallographic information of protein and the center of the grid box and the dimension during docking simulations using AutoDock Vina; Table S4: Binding energy and binding interactions of meclofenamic acid, arachidonic acid and ICSB docked against human and murine COX-2 protein; Figure S1: Superposition of bound ligand for the validation of docking protocol; Figure S2: Molecular docking analysis of arachidonic acid with X-ray crystallographic structure of human COX-2 protein (1CVU).; Figure S3: Molecular docking analysis meclofenamic acid with X-ray crystallographic structure of murine COX-2 protein (PDB: 5IKQ).

## Abbreviations

ANOVA	Analysis of variance
CNS	Central nervous system
ELISA	Enzyme-linked immunosorbent assay
ERK	Extracellular signal-regulated kinase
ICR	Institute of Cancer Research
MAPK	Mitogen-activated protein kinase
PDB	Protein Data Bank
