# Supplementary material for: Attenuation of Inflammatory Symptoms by Icariside B2 in Carrageenan and LPS-Induced Inflammation Models via Regulation of MAPK/NF-κB Signaling Cascades
Source: Biomolecules. 2020 Jul 11;10(7):1037. doi: 10.3390/biom10071037 (PMC7408066; doi:10.3390/biom10071037)
Supplement: Supplementary file 1 [file biomolecules-10-01037-s001.pdf]

Table S1. List of the primer sets used in the study.

Gene Name		Sequences
iNOS	*forward*	*GGCTGTCAGAGCCTCGTGGC*
	reverse	*CCCTTCCGAAGTTTCTGGCA*
COX2	*forward*	*AACACAGCTACGAAAACC*
	reverse	*CACAGTATGATGTAACAGT*
TNFα	*forward*	*GGCAGGTCTACTTTGGAGTCA*
	reverse	*ACATTCGAGGCTCCAGTGAAT*
IL-1β	*forward*	*ATGGCAACTGTTCCTGAACTC*
	reverse	*CAGGACAGGTATAGATTCTTT*
IL6	*forward*	*GAGGATACCACTCCCAACAGA*
	reverse	*AAGTGCATCATCGTTGTTCATA*
Gapdh	*forward*	*TTGTGATGGGTGTGAACCAC*
	reverse	*ACACATTGGGGGTAGGAACA*

Table S2. List of the primary antibodies used in the study.

Name	Catalog no.	Company	Antigen	Host
Anti-iNOS	MAB9502	R&D systems	iNOS	Mouse
Anti-COX2	AF4198	R&D systems	COX2	Goat
Anti-p-IκB-α	sc-8404	Santa Cruz Biotechnology, Inc.	IκB-α	Mouse
Anti-IκB-α	sc-373893	Santa Cruz Biotechnology, Inc.	IκB-α	Mouse
Anti-NF-κB (p65)	BS1254	Bioworld Technology, Inc.	NF-κB (p65)	Rabbit
Anti-Histone H3	BS1405	Bioworld Technology, Inc.	Histone H3	Rabbit
Anti-p-p38	sc-166182	Santa Cruz Biotechnology, Inc.	p38	Mouse
Anti-p38	BS3567	Bioworld Technology, Inc.	p38	Rabbit
Anti-p-ERK1/2	sc-7383	Santa Cruz Biotechnology, Inc.	ERK	Mouse
Anti-ERK1/2	BS 6472	Bioworld Technology, Inc.	ERK	Rabbit
Anti-p-JNK	BS 4322	Bioworld Technology, Inc.	JNK	Rabbit
Anti-JNK	sc-7345	Santa Cruz Biotechnology, Inc.	JNK	Mouse
Anti- βactin	Sc-47778	Santa Cruz Biotechnology, Inc.	β-actin	Mouse

Table S3. The X-ray crystallographic information of protein and the center of the grid box and the dimension during docking simulations using AutoDock Vina.

PDB ID	Source	Resolution	Co-crystallized inhibitor	Grid box			
				Center		Dimension	
5IKQ	Homo sapiens	2.41 Å	Meclofenamic Acid	x	27.34	x	25
				y	45.72	y	25
				z	19.61	z	25
1CVU	Mus musculus	2.40 Å	Arachidonic Acid	x	28.37	x	25
				y	29.09	y	25
				z	40.76	z	25

Table S4. Binding energy and binding interactions of meclofenamic acid, arachidonic acid and ICSB docked against human and murine COX-2 protein.

PDB ID	Ligand name	Binding energy (kcal/mol)	Binding interactions		
			Hydrogen bond interaction residue	Bond length (Å)	Hydrophobic bond interaction residue
5IKQ	Meclofenamic acid	-9.0	Ser530	3.13	Ala527, Gly526, Leu352, Leu531, Met522, Ser353, Trp387, Tyr 348, Tyr385, and Val349
	ICSB	-8.0	Arg222	3.31	Gln203, Gln289, His214, His386, His388, Lys211, Phe210, and Val291
				3.14	
			Asn382	3.08	
			Thr206	2.77	
			Tyr385	2.71	
				3.01	
			Trp387	2.79	
1CVU	Arachidonic acid	-7.9	Leu531	3.15	Ala527, Gly526, Leu352, Leu359, Leu531, Met113, Met522, Phe 381, Phe518, Ser353, Ser530, Trp387, Tyr 355, Tyr385, Val166, Val349, and Val523
	ICSB	-7.4	Ala199	2.80	Ala 202, Gln203, His207, His214, His386, His388, Leu390, Leu391, and Val447
			Thr212	3.34	
			Thr206	2.77	
			Trp387	2.98	

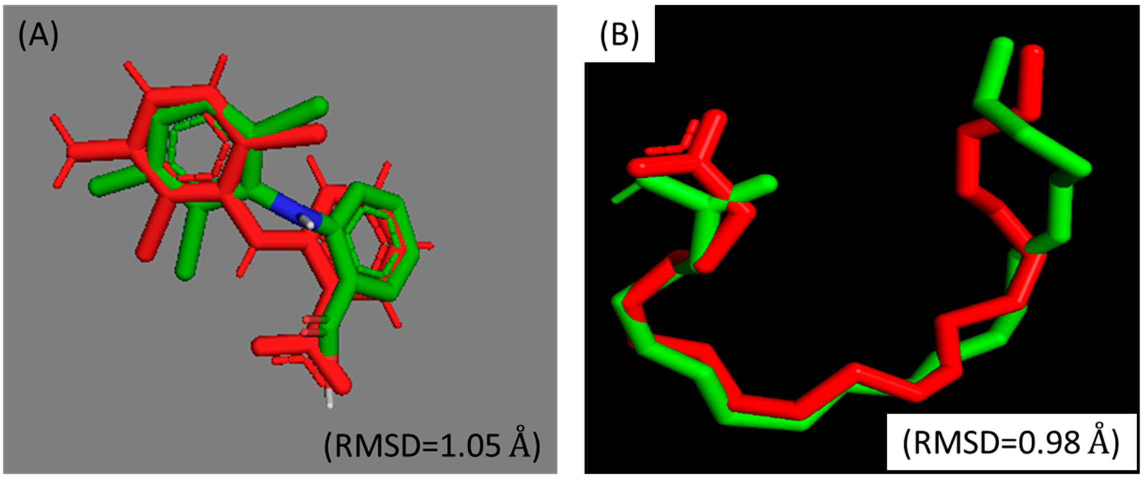

Figure S1. Superposition of bound ligand for the validation of docking protocol. Superposition of the (**A**) best docked structure (green) and crystallographic structure for the human COX-2 protein and meclofenamic acid complex (red) (PDB accession no. 5IKQ) and (**B**) best docked structure (green) and crystallographic structure for the murin COX-2 protein and arachidonic acid complex (red) (PDB accession no. 1CVU).

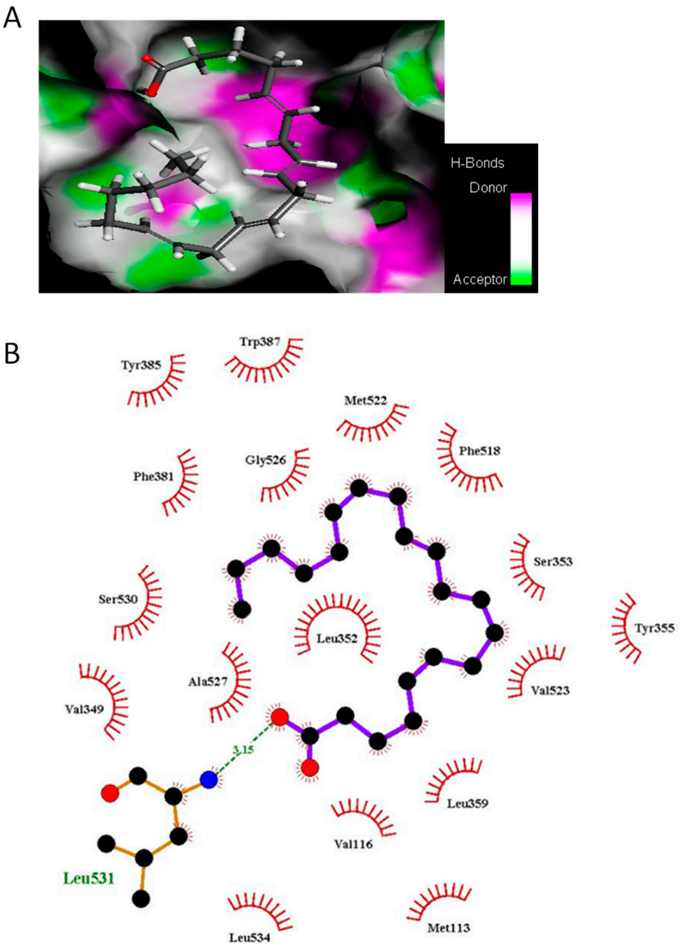

Figure S2. Molecular docking analysis of arachidonic acid with X-ray crystallographic structure of human COX-2 protein (1CVU). Bonding interaction between original ligand (arachidonic acid) (**A** and **B**) human COX-2 protein (1CVU). The 3D and 2D binding conformation were visualized as diagrams using Discovery Studio Visualization version 4.5 and LigPlot viewer.

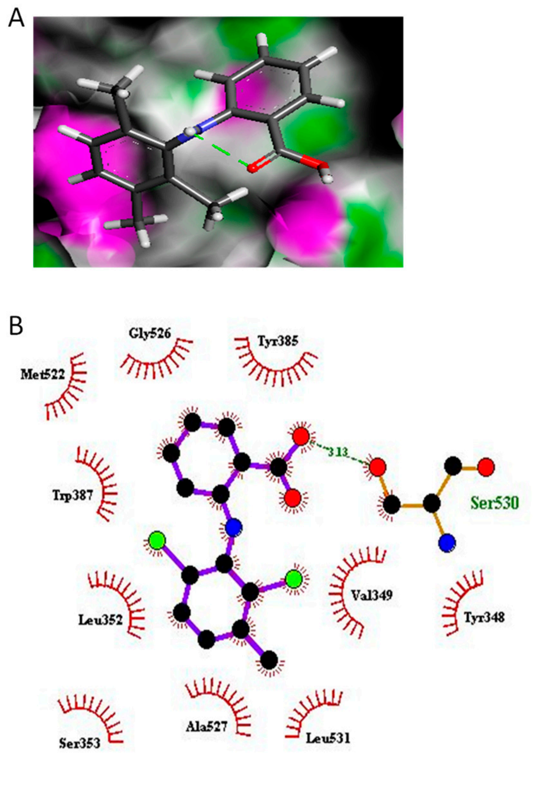

Figure S3. Molecular docking analysis meclofenamic acid with X-ray crystallographic structure of murine COX-2 protein (PDB: 5IKQ). Bonding interaction between original ligand (meclofenamic acid) (**A** and **B**) and murine COX-2 protein (PDB: 5IKQ). The 3D and 2D binding conformation were visualized as diagrams using Discovery Studio Visualization version 4.5 and LigPlot viewer.
